# Intuitionistic Fuzzy Hierarchical Multi-Criteria Decision Making for Evaluating Performances of Low-Carbon Tourism Scenic Spots

**DOI:** 10.3390/ijerph17176259

**Published:** 2020-08-28

**Authors:** Xuan Yang, Zhou-Jing Wang

**Affiliations:** 1 Dongfang College, Zhejiang University of Finance & Economics, Haining 314408, China; yx_321@zufe.edu.cn; 2 School of Information, Zhejiang University of Finance & Economics, Hangzhou 310018, China

**Keywords:** low-carbon economy, low-carbon tourism scenic spot, multi-criteria decision making, intuitionistic fuzzy preference relation, evaluation

## Abstract

Low-carbon tourism is an effective solution to cope with the goal conflict between developing tourist economy and responding to carbon emission reduction and ecological environment protection. Tourism scenic spots are important carriers of tourist activities and play a crucial role in low-carbon tourism. There are multiple factors affecting the low-carbon performance of a tourism scenic spot, and thus the performance evaluation and ranking of low-carbon tourism scenic spots can be framed as a hierarchical multi-criteria decision making (MCDM) problem. This paper develops a novel method to tackle hierarchical MCDM problems, in which the importance preferences of criteria over the decision goal and sub-criteria with respect to the upper-level criterion are provided by linguistic-term-based pairwise comparisons and the assessments of alternatives over each of sub-criteria at the lowest level are furnished by positive interval values. The linguistic-term-based pairwise comparison matrices are converted into intuitionistic fuzzy preference relations and an approach is developed to obtain the global importance weights of the lowest level sub-criteria. A multiplicatively normalized intuitionistic fuzzy decision matrix is established from the interval-value-based assessments of alternatives and a method is proposed to determine the intuitionistic fuzzy value based comprehensive scores of alternatives. A case study is offered to illustrate how to build a performance evaluation index system of low-carbon tourism scenic spots located at Zhejiang Province of China and show the use of the proposed intuitionistic fuzzy hierarchical MCDM method.

## 1. Introduction

With the increasing challenge on climate change, low-carbon economy has become a consensual solution for coping with global warming and preserving ecological environment [1]. Its core is to obtain a higher quality economic development mode with minimizing energy consumption, less environmental pollution, and low carbon emission [2]. In 2006, China had stated that Chinese economy walks the low-carbon development pattern. China declared its carbon emission reduction goal in the 2008 Climate Change Conference of United Nation in Copenhagen. Furthermore, China expects that by the end of 2030, the carbon intensity (i.e., the emission of Unit GDP CO_2_) will be reduced by between 60% and 65% from 2005. On the other hand, tourism has been recognized as an advantageous industry for the economic development and identified as a remarkable carbon emission producer [3,4]. Peeters and Dubois [5] indicated that tourism industry was responsible for 4.4% of the 2005 global carbon emission. They also predicted that with continuing the traditional tourism mode, the carbon emission caused by tourism industry would be increased by an annually average rate of 3.2% up to 2035. Zha et al. [6] showed that the carbon emission of tourism in Hubei Province of China skyrocketed from 6,340,302 tons in 2007 to 23,939,851 tons in 2013. Sun [7] stressed that it is necessary to reduce the tourism carbon emission at a rate of at least 3% per year for eliminating dangerous climate change. As a result, low-carbon tourism has been determined as a sustainable and green tourism development mode and plays an important application of low-carbon economy [8,9].

Many scholars have paid attention to studies on tourism carbon emissions and low-carbon tourism. For instance, Huang and Deng [10] indicated that low-carbon tourism is an important development direction and established a model to measure the development of low-carbon tourism in China. Gössling et al. [11] discussed the tourism food management from the viewpoint of tourists’ carbon footprint and concluded that the tourism carbon emission can be reduced by the effective food management. Durbarry and Seetanah [12] studied the dynamic relationship between climate change and tourism carbon emissions. Gössling et al. [13] showed that the difference between the tourism carbon intensity and the carbon reduction target was actually growing by the end of 2010, and indicated that the tourism revenue can be optimized by developing low-carbon tourism. Kuo and Dai [14] explored the important factors affecting tourists’ behavior of low-carbon tourism, and established a predictive model of the low-carbon tourism behavior in Taiwan. Tang et al. [15] investigated the effects of tourism activities, accommodation and transportation on the total tourism carbon emission. Sun et al. [16] proposed a multi-criteria decision analysis based framework managing tourism carbon emissions through the combinatorial optimization of tourism demands.

There are a number of studies in evaluating the performance and efficiency of low-carbon tourism systems. Cheng et al. [17] established a 4-level index system consisting of 27 lowest-level sub-criteria concerned with eco-environment, tourist facilities, management and participant attitudes to evaluate the low-carbon performance of tourism attractions. They utilized the commonly used multi-criteria decision making (MCDM) method called analytic hierarchy process (AHP) to obtain real-valued importance weights of criteria and sub-criteria, and gave a case study of the low-carbon performance evaluation for the Xixi National Wetland located at Hangzhou of China. Cho et al. [18] constructed a framework of Taiwan’s low-carbon tourism evaluation indicators concerning with travel agencies, transportation, hotels and accommodation, food service, tourism scenic spots, and local communities, and used the fuzzy AHP to derive real-valued weights of criteria and sub-criteria. Zhang [19] developed a triangular fuzzy Delphi-analytic network process model to evaluate regional low-carbon tourism strategies, and applied it to determine priority weights of low-carbon tourism strategies in China’s Chengguan District of Lhasa. Zha et al. [6] used the data envelopment analysis method to evaluate the economic development efficiency of low-carbon tourism cities. Lin and Wang [20] proposed a group MCDM model with linguistic assessments and incomplete criterion weight information, and applied it in evaluating and ranking low-carbon tourism destinations. Liu et al. [21] devised an evaluation model of low-carbon tourism scenic spots by means of combining the best-worst MCDM method [22] with Dempster-Shafer evidence theory and VIKOR method, in which triangular intuitionistic fuzzy numbers are used to characterize experts’ judgments for the importance of criteria and sub-criteria, and to express experts’ assessments of alternatives on each of the sub-criteria at the lowest-level. Zhang et al. [23] put forward an intuitionistic multiplicative prioritization method and combined it with the ordinal regression method [24] to evaluate and rank low-carbon tourism destinations, where the importance weights of criteria are directly derived from intuitionistic multiplicative preference relations and experts’ assessments of alternatives on each of criteria are provided by positive real values or intuitionistic multiplicative numbers. However, any of the aforesaid evaluation models is unable to be used to solve hierarchical evaluation problems, where the assessments of evaluated alternatives over the lowest level sub-criteria are provided by different dimension interval values obtained from statistical tables and questionnaires.

Tourism scenic spots are pivotal carriers of tourism activities and their low-carbon performance and efficiency evaluation is conducive to the development strategy formulation of low-carbon tourism. The aforesaid literature review reveals that such an evaluation index system involves many low-carbon economy-, environment-, and management-based criteria and sub-criteria whose importance weights are often without past data. Hence, an important stage in evaluating performances of low-carbon tourism scenic spots is to build an evaluation index based hierarchical structure and determine importance weights of criteria and sub-criteria from experts’ pairwise comparison based judgments [18,19,21]. Considering the hesitancy of experts’ judgements, this study employs intuitionistic fuzzy preference relations (IFPRs) [25] to characterize experts’ linguistic term based pairwise comparison results and uses the intuitionistic fuzzy priority method [26] to obtain local importance weights of criteria and sub-criteria, which are expressed as multiplicatively normalized intuitionistic fuzzy weights (MNIFWs). On the other hand, in this study, the assessments of alternatives over sub-criteria at the lowest level are collected from statistical tables and questionnaires and not obtained from experts’ pairwise comparisons. This implies that it is necessary to obtain the global importance weights of sub-criteria at the lowest level. Therefore, this paper devises a scale conversion between linguistic terms and intuitionistic fuzzy values (IFVs) and develops a novel approach to aggregate local importance weights of criteria and sub-criteria into the global importance weights of the lowest level sub-criteria in an evaluation index system.

Another important stage in evaluating performances of low-carbon tourism scenic spots is to obtain the comprehensive scores of alternatives [17,19,22,23,24]. In this study, the assessments of alternatives over the lowest level sub-criteria are characterized by positive interval values whose measuring units are not uniform. On the other hand, since the criteria importance weights are expressed by MNIFWs, it is natural to expect that the obtained comprehensive scores are MNIFWs. Few of the existing MCDM methods can be employed to solve such decision scenarios. To overcome this issue, this paper proposes a method to construct multiplicatively normalized intuitionistic fuzzy decision matrix from the interval-value-based assessments of alternatives. An approach including a maximization optimization model is established to obtain MNIFW based comprehensive scores of alternatives.

The remainder of this paper is framed in the following way. Section 2 offers basic knowledges on intuitionistic fuzzy sets, IFPRs and computational formulas of obtaining MNIFWs from IFPRs. Section 3 develops an intuitionistic fuzzy hierarchical MCDM model. In Section 4, an evaluation index system of low-carbon tourism scenic spots is established and an example is furnished to illustrate how to apply the developed model in evaluating low-carbon tourism scenic spots. Some concluding remarks are followed in Section 5.

## 2. Preliminaries

Let Z be a fixed set, an intuitionistic fuzzy set [27] $\tilde{A}$ on Z is characterized as

(1) $$\tilde{A}=\left\{<z,\xi_{\tilde{A}}(z),\eta_{\tilde{A}}(z)>|z\in Z\right\}$$

where $\xi_{\tilde{A}}:Z\to [0,1]$ and $\eta_{\tilde{A}}:Z\to [0,1]$ are the membership function and the non-membership function of element z to $\tilde{A}$, respectively, and inequality $\xi_{\tilde{A}}(z)+\eta_{\tilde{A}}(z)\leq 1$ holds true for every z∈Z.

It is clear that the intuitionistic fuzzy set $\tilde{A}$ becomes an ordinary fuzzy set if $\xi_{\tilde{A}}(z)+\eta_{\tilde{A}}(z)=1$ for all z∈Z. In this case, the non-membership degree $\eta_{\tilde{A}}(z)$ can be immediately obtained from its membership degree $\xi_{\tilde{A}}(z)$ for every z∈Z.

For an intuitionistic fuzzy set $\tilde{A}$ and a given z∈Z, a pair $<\xi_{\tilde{A}}(z),\eta_{\tilde{A}}(z)>$ is said an IFV [25]. For convenience, the pair $<\xi_{\tilde{A}}(z),\eta_{\tilde{A}}(z)>$ is simply denoted as <ξ,η>, where 0≤ξ,η≤1 and ξ+η≤1.

For an IFV $\tilde{\alpha }=<\xi ,\eta >$ with 0<ξ,η≤1, its hesitancy ratio is measured [28] as

(2) $$H(\tilde{\alpha })=\frac{\left(1-\xi )(1-\eta \right)}{\xi \eta }.$$

It is obvious that $H(\tilde{\alpha })\geq 1$. The bigger the ratio $H(\tilde{\alpha })$, the greater the hesitancy intensity of the IFV $\tilde{\alpha }$. If $H(\tilde{\alpha })=1$, then μ+v=1, revealing that $\tilde{\alpha }$ reduces to an exact value ξ. Additionally, it is easy from (2) to confirm that $H({\tilde{\alpha }}^{c})=H(\tilde{\alpha })$, where ${\tilde{\alpha }}^{c}=<\eta ,\xi >$ is the reciprocal of $\tilde{\alpha }$.

Let $X=\{x_{1},x_{2},\ldots ,x_{n}\}$ be a collection of objects, where $x_{i}$ could be an alternative or a criterion. An IFPR [25] on X is characterized by a square matrix $\tilde{R}={\left({\tilde{r}}_{ij}\right)}_{n\times n}={\left(<\xi_{ij},\eta_{ij}>\right)}_{n\times n}$, where ${\tilde{r}}_{ij}=<\xi_{ij},\eta_{ij}>$ is an IFV indicating the intuitionistic fuzzy preference of object $x_{i}$ over $x_{j}$, and

(3) $$0\leq \xi_{ij}+\eta_{ij}\leq 1,\;\xi_{ii}=\eta_{ii}=0.5,\;\xi_{ij}=\eta_{ji},\;i,j=1,2,\ldots ,n.$$

Wang [26] showed that any 2×2 IFPR is consistent and introduced a consistency concept for IFPRs with order n≥3.

An IFPR $\tilde{R}={\left({\tilde{r}}_{ij}\right)}_{n\times n}={\left(<\xi_{ij},\eta_{ij}>\right)}_{n\times n}$ (n≥3) is said a consistent IFPR [26] if

(4) $$\mu_{ij}=\frac{\delta_{k}\xi_{ik}\eta_{kj}}{\delta_{k}\xi_{ik}\eta_{kj}+(1-\delta_{k})(1-\xi_{ik})(1-\eta_{kj})},\;i,j,k=1,2,\ldots ,n,\;i\neq j\neq k,$$

where $\delta_{k}$ is defined as (A1) in Appendix A.

In order to measure inconsistency and check acceptable consistency for IFPRs, Wang [26] designed a consistency index $CI\left(\tilde{R}\right)$ whose computational formula is provided as (A2) in Appendix A.

Let $t_{c}$ ($0<t_{c}<1$) be a threshold of acceptable consistency, then $\tilde{R}$ is said an acceptably consistent IFPR when $CI\left(\tilde{R}\right)\leq t_{c}$. If $CI\left(\tilde{R}\right)=0$, then $\tilde{R}$ is a consistent IFPR.

An acceptable IFPR should both possess acceptable consistency and acceptable hesitancy. In other words, an IFPR $\tilde{R}={\left({\tilde{r}}_{ij}\right)}_{n\times n}={\left(<\xi_{ij},\eta_{ij}>\right)}_{n\times n}$ is said to be acceptable [26] if $\tilde{R}$ is acceptably consistent and $\theta_{i}\leq t_{h}$ for all i=1,2,…,n, where $t_{h}$ ($t_{h}\geq 1$) is a threshold of acceptable hesitancy and $\theta_{i}$ is defined as (A3) in Appendix A.

Let $\tilde{W}={\left({\tilde{w}}_{1},{\tilde{w}}_{2},\ldots ,{\tilde{w}}_{n}\right)}^{T}$ be an intuitionistic fuzzy weight vector, where ${\tilde{w}}_{i}=<\mu_{i},v_{i}>$ for i=1,2,…,n, then $\tilde{W}$ is said to be multiplicatively normalized [26] if

(5) $$\prod_{i=1}^{n}\frac{\mu_{i}(1-v_{i})}{v_{i}(1-\mu_{i})}=1.$$

Wang [26] developed computational Formulas (A4) and (A5) given in Appendix A for obtaining optimized MNIFWs ${\tilde{w}}_{i}^{*}=<\mu_{i}^{*},v_{i}^{*}>$ (i=1,2,…,n) from an acceptable IFPR $\tilde{R}={\left({\tilde{r}}_{ij}\right)}_{n\times n}={\left(<\xi_{ij},\eta_{ij}>\right)}_{n\times n}$.

## 3. An Intuitionistic Fuzzy Hierarchical Multi-Criteria Decision Making Method

### 3.1. The Hierarchical Structure of Criteria in an Evaluation System

**Figure 1:**
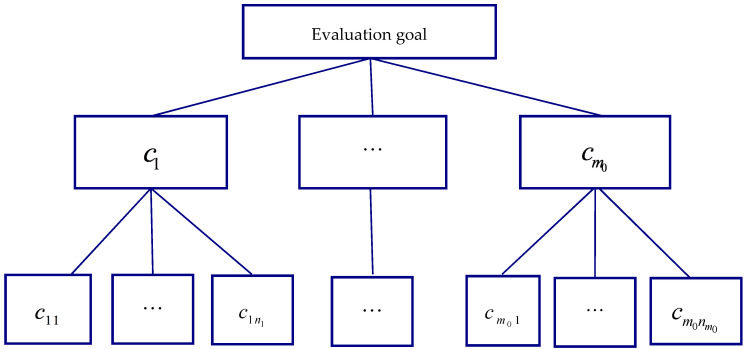
A hierarchical structure.

A complex evaluation problem often involves multiple criteria and sub-criteria. In order to easily obtain importance weights of criteria, it is necessary to establish a hierarchical structure, in which the evaluation goal is at the highest level, criteria and sub-criteria are at the middle levels or at the lowest level. Figure 1 graphically illustrates a 3-level hierarchical structure, where criteria $c_{1},c_{2},\ldots ,c_{m_{0}}$ are at the middle level and sub-criteria $c_{j1},c_{j2},\ldots ,c_{jn_{j}}$ relating with the upper-level criterion $c_{j}$ are at the lowest level for each $j=1,2,\ldots ,m_{0}$.

It should be noted that this hierarchical structure differs from that of AHP [29] or the intuitionistic fuzzy AHP [26] whose lowest level objects are alternatives. This is because the assessment data of alternatives over the lowest level criteria is not provided by experts’ pairwise comparisons in this study.

### 3.2. Determining Importance Weights of Criteria

To obtain important weights of criteria/sub-criteria, the paired comparison method is used to elicit the preference between any two criteria/sub-criteria at one level with respect to the same upper-level criterion or goal. In this paper, paired comparisons are made with the help of bipolar linguistic term scales, and are characterized by IFVs. The linguistic term scales and their corresponding intuitionistic fuzzy conversion scales are devised as shown in Table 1.

It is clear that the hierarchical structure of an evaluation problem can be viewed as a tree whose children nodes represent criteria or sub-criteria. Based on the scales listed in Table 1, for q (q≥2) criteria/sub-criteria with the same parent, an q×q IFPR is obtained by comparing each pair of the criteria/sub-criteria. If this IFPR is unacceptable, then it is asked to be revised; otherwise, the optimized local importance weights of these criteria or sub-criteria are determined by using the two computational Formulas (A4) and (A5) in Appendix A. For any single criterion/sub-criterion with no sibling, its local importance weight is set to be <0.5, 0.5>.

Once local importance weights of all criteria/sub-criteria in children nodes have been obtained, the next step is to determine a global importance weight for each criterion/sub-criterion in leaf nodes with respect to the evaluation goal.

Obviously, there always exists a path from the root node to any leaf node. Let h be the depth of the tree, and $c_{j}^{h}$ (j=1,2,…,m) be criteria or sub-criteria in leaf nodes, then a global importance weight ${\tilde{w}}_{c_{j}^{h}}^{\#}=<\mu_{c_{j}^{h}}^{\#},v_{c_{j}^{h}}^{\#}>$ of criterion/sub-criterion $c_{j}^{h}$ is determined as

(6) $$\mu_{c_{j}^{h}}^{\#}=\frac{\prod_{k=2}^{h}{\left(\mu_{c_{j}^{k}}\right)}^{1/n_{c_{j}^{k}}}}{\prod_{k=2}^{h}{\left(\mu_{c_{j}^{k}}\right)}^{1/n_{c_{j}^{k}}}+\prod_{k=2}^{h}{\left(1-\mu_{c_{j}^{k}}\right)}^{1/n_{c_{j}^{k}}}},\;j=1,2,\ldots ,m$$

(7) $$v_{c_{j}^{h}}^{\#}=\frac{\prod_{k=2}^{h}{\left(v_{c_{j}^{k}}\right)}^{1/n_{c_{j}^{k}}}}{\prod_{k=2}^{h}{\left(v_{c_{j}^{k}}\right)}^{1/n_{c_{j}^{k}}}+\prod_{k=2}^{h}{\left(1-v_{c_{j}^{k}}\right)}^{1/n_{c_{j}^{k}}}},\;j=1,2,\ldots ,m$$

where $c_{j}^{k}$ is the *k*th level criterion/sub-criterion in the path from the root node to the leaf node $c_{j}^{h}$, and ${\tilde{w}}_{c_{j}^{k}}=<\mu_{c_{j}^{k}},v_{c_{j}^{k}}>$ is the local importance weight of $c_{j}^{k}$ for j=1,2,…,m,k=2,3,…,h and $n_{c_{j}^{k}}$ is defined as

(8) $$n_{c_{j}^{k}}=\{\begin{array}{cc} 1, & k=h\\ q_{c_{j}^{k}}, & k\neq h \end{array},\;k=2,3,\ldots ,h$$

where $q_{c_{j}^{k}}$ is the number of leaf nodes in the sub-tree with root node $c_{j}^{k}$ for all j=1,2,…,m,k=2,3,…,h.

It is noted that there does not exist an approach obtaining the above global importance weights of the lowest level criteria/sub-criteria in the current literature. The intuitionistic fuzzy AHP based MCDM methods needs only to elicit local importance weights of criteria and sub-criteria [26,30] while other intuitionistic fuzzy MCDM methods focus mainly on decision problems with single-layer structures [31,32].

**Theorem** **1.** *Let*${\tilde{w}}_{c_{j}^{h}}^{\#}=<\mu_{c_{j}^{h}}^{\#},v_{c_{j}^{h}}^{\#}>$*be the global importance weight defined by (6) and (7) for every*j=1,2,…,m*, then intuitionistic fuzzy weights*${\tilde{w}}_{c_{j}^{h}}^{\#}=<\mu_{c_{j}^{h}}^{\#},v_{c_{j}^{h}}^{\#}>$*(*j=1,2,…,m*) are multiplicatively normalized, i.e.,*$\prod_{j=1}^{m}\frac{\mu_{c_{j}^{h}}^{\#}(1-v_{c_{j}^{h}}^{\#})}{v_{c_{j}^{h}}^{\#}(1-\mu_{c_{j}^{h}}^{\#})}=1$.

**Proof.**  It is obvious that local importance weights of criteria/sub-criteria with the same parent are multiplicatively normalized. In other words, (5) holds for local importance weights of criteria/sub-criteria with the same parent. On the other hand, as per (6) and (7), one has

$$\prod_{j=1}^{m}\frac{\mu_{c_{j}^{h}}^{\#}(1-v_{c_{j}^{h}}^{\#})}{v_{c_{j}^{h}}^{\#}(1-\mu_{c_{j}^{h}}^{\#})}=\prod_{k=2}^{h}\prod_{l=1}^{r_{k}}\frac{\mu_{l}^{k}(1-v_{l}^{k})}{v_{l}^{k}(1-\mu_{l}^{k})}=1$$

where $r_{k}$ is the number of nodes at the *k*th level for k=2,3,…,h and ${\tilde{w}}_{l}^{k}=<\mu_{l}^{k},v_{l}^{k}>$ is the local importance weight of the criterion/sub-criterion corresponding to the *l*th node at the *k*th level for $l=1,2,\ldots ,r_{k},k=2,3,\ldots ,h$. Therefore, ${\tilde{w}}_{c_{j}^{h}}^{\#}$ (j=1,2,…,m) are multiplicatively normalized. □

Theorem 1 indicates that an MNIFW scheme is determined for the criteria or sub-criteria at the lowest level.

### 3.3. Establishing an Intuitionistic Fuzzy Decision Matrix

Let $C^{h}=\{c_{1}^{h},c_{2}^{h},\ldots ,c_{m}^{h}\}$ be the set of m criteria/sub-criteria at the lowest level, and the assessment of alternative $x_{i}$ over criterion/sub-criterion $c_{j}^{h}$ (j=1,2,…,m) be provided by a positive interval ${\bar{a}}_{ij}=[a_{ij}^{-},a_{ij}^{+}]$ for every i=1,2,…,n, then an IFPR ${\tilde{R}}_{c_{j}^{h}}^{\&}={\left(<\xi_{st}^{\&},\eta_{st}^{\&}>\right)}_{n\times n}$ is generated by the following formulas:

(9) $$\begin{array}{l} \xi_{st}^{\&}=\\ \{\begin{array}{ll} 0.5, & s=t\\ \frac{a_{sj}^{-}}{a_{sj}^{-}+a_{tj}^{+}}, & s\neq t\;\mathrm{and}\;c_{j}^{h}\;\mathrm{is}\;a\;\mathrm{benefit}\;\mathrm{criterion}\\ \frac{a_{tj}^{-}}{a_{tj}^{-}+a_{sj}^{+}}, & s\neq t\;\mathrm{and}\;c_{j}^{h}\;\mathrm{is}\;a\;\cos t\;\mathrm{criterion}\; \end{array} \end{array},\;s,t=1,2,\ldots ,n$$

(10) $$\begin{array}{l} \eta_{st}^{\&}=\\ \{\begin{array}{ll} 0.5, & s=t\\ \frac{a_{tj}^{-}}{a_{tj}^{-}+a_{sj}^{+}}, & s\neq t\;\mathrm{and}\;c_{j}^{h}\;\mathrm{is}\;a\;\mathrm{benefit}\;\mathrm{criterion}\\ \frac{a_{sj}^{-}}{a_{sj}^{-}+a_{tj}^{+}}, & s\neq t\;\mathrm{and}\;c_{j}^{h}\;\mathrm{is}\;a\;\cos t\;\mathrm{criterion}\; \end{array} \end{array},\;s,t=1,2,\ldots ,n.$$

By (A2), one has $CI\left({\tilde{R}}_{c_{j}^{h}}^{\&}\right)=0$. This shows that ${\tilde{R}}_{c_{j}^{h}}^{\&}$ is a consistent IFPR. If ${\tilde{R}}_{c_{j}^{h}}^{\&}$ has no acceptable hesitancy, then the interval assessments ${\bar{a}}_{ij}$ (i=1,2,…,n) are asked to be modified; otherwise, by (A4) and (A5), an MNIFW vector is obtained from ${\tilde{R}}_{c_{j}^{h}}^{\&}$ and is denoted by ${\tilde{W}}_{c_{j}^{h}}^{\&}={\left({\tilde{w}}_{1c_{j}^{h}}^{\&},{\tilde{w}}_{2c_{j}^{h}}^{\&},\ldots ,{\tilde{w}}_{nc_{j}^{h}}^{\&}\right)}^{T}$, where ${\tilde{w}}_{ic_{j}^{h}}^{\&}=<\mu_{ic_{j}^{h}}^{\&},v_{ic_{j}^{h}}^{\&}>$ for every i=1,2,…,n. Based on ${\tilde{W}}_{c_{j}^{h}}^{\&}$, the IFV based assessment of alternative $x_{i}$ over criterion $c_{j}^{h}$ is determined as ${\tilde{a}}_{ij}=<\phi_{ij},\varphi_{ij}>=<\mu_{ic_{j}^{h}}^{\&},v_{ic_{j}^{h}}^{\&}>$ for every i=1,2,…,n. Obviously, the IFV based vector ${\left({\tilde{a}}_{1j},{\tilde{a}}_{2j},\ldots ,{\tilde{a}}_{nj}\right)}^{T}$ is multiplicatively normalized for each j=1,2,…,m. Thus, a multiplicatively normalized intuitionistic fuzzy decision matrix is constructed as $\tilde{A}={\left({\tilde{a}}_{ij}\right)}_{n\times m}$.

It is noted that the normalized intuitionistic fuzzy decision matrix establishing method herein differs from any existing method in the literature. Firstly, the IFPRs ${\tilde{R}}_{c_{j}^{h}}^{\&}$ (j=1,2,…,m) are not provided by decision makers. They are obtained from the interval assessments ${\bar{a}}_{ij}$ (i=1,2,…,n, j=1,2,…,m) by using (9) and (10). Secondly, the intuitionistic fuzzy elements in $\tilde{A}$ are multiplicatively normalized by determining intuitionistic fuzzy weights of ${\tilde{R}}_{c_{j}^{h}}^{\&}$ (j=1,2,…,m).

### 3.4. Obtaining Comprehensive Scores of Evaluated Alternatives

Once the global importance weight scheme of criteria or sub-criteria in $C^{h}$ and the normalized intuitionistic fuzzy decision matrix $\tilde{A}={\left({\tilde{a}}_{ij}\right)}_{n\times m}={\left(<\phi_{ij},\varphi_{ij}>\right)}_{n\times m}$ have been determined, the next step in an evaluation process is to aggregate the IFV based assessments together with the global importance weights of criteria/sub-criteria in $C^{h}$ into comprehensive scores of evaluated alternatives. Because the global importance weights ${\tilde{w}}_{c_{j}^{h}}^{\#}=<\mu_{c_{j}^{h}}^{\#},v_{c_{j}^{h}}^{\#}>$ (j=1,2,…,m) determined in Section 3.2 are MNIFWs and the elements in $\tilde{A}$ are all IFVs, it is difficult to employ an existing IFV aggregation operator for obtaining the comprehensive score of an evaluated alternative from $\tilde{A}$.

If the importance of criterion/sub-criterion $c_{j}^{h}$ is determined to be a positive real weight $\omega_{j}$ for every j=1,2,…,m, where $\sum_{j=1}^{m}\omega_{j}=1$, then an IFV based comprehensive score can be obtained as

(11) $${\tilde{s}}_{x_{i}}=<\mu_{x_{i}},v_{x_{i}}>=\langle \frac{\prod_{j=1}^{m}{\left(\phi_{ij}\right)}^{\omega_{j}}}{\prod_{j=1}^{m}{\left(\phi_{ij}\right)}^{\omega_{j}}+\prod_{j=1}^{m}{\left(1-\phi_{ij}\right)}^{\omega_{j}}},\frac{\prod_{j=1}^{m}{\left(\varphi_{ij}\right)}^{\omega_{j}}}{\prod_{j=1}^{m}{\left(\varphi_{ij}\right)}^{\omega_{j}}+\prod_{j=1}^{m}{\left(1-\varphi_{ij}\right)}^{\omega_{j}}}\rangle ,\;i=1,2,\ldots ,n$$

Clearly, the IFV ${\tilde{s}}_{x_{i}}$ is isomorphic to an IFV ${\tilde{s}}_{x_{i}}^{\&}=\langle \sum_{j=1}^{m}\left(\phi_{ij}\omega_{j}\right),\sum_{j=1}^{m}\left(\varphi_{ij}\omega_{j}\right)\rangle$. The greater the IFV ${\tilde{s}}_{x_{i}}^{\&}$, the larger the IFV ${\tilde{s}}_{x_{i}}$ and the more the superiority of alternative $x_{i}$. Since $<\phi_{ij},\varphi_{ij}>$ reflects the satisfaction and non-satisfaction degrees of the alternative $x_{i}$ over the criterion $c_{j}^{h}$, it is necessary to determine real weights $\omega_{j}$ (j=1,2,…,m) from MNIFWs ${\tilde{w}}_{c_{j}^{h}}^{\#}$ (j=1,2,…,m) such that such real weights maximize ${\tilde{s}}_{x_{i}}^{\&}$ for every i=1,2,…,n. Therefore, a goal programming model is built as

(12) $$\begin{array}{l} \max\;Z_{i}=\sum_{j=1}^{m}\left(q_{ij}\omega_{j}\right)\\ s.t.\{\begin{array}{ll} \sum_{j=1}^{m}\omega_{j}=1, & \\ \frac{\mu_{c_{j}^{h}}^{\#}}{1-\mu_{c_{j}^{h}}^{\#}}\leq \frac{\omega_{j}}{{\left(\prod_{l=1}^{m}\omega_{l}\right)}^{1/m}}\leq \frac{1-v_{c_{j}^{h}}^{\#}}{v_{c_{j}^{h}}^{\#}}, & j=1,2,\ldots ,m\\ \phi_{ij}\leq q_{ij}\leq 1-\varphi_{ij}, & j=1,2,\ldots ,m \end{array} \end{array},\;i=1,2,\ldots ,n$$

where the first two line constraints ensure that $\omega_{j}$ (j=1,2,…,m) are additively normalized and $\omega_{j}/{\left(\prod_{l=1}^{m}\omega_{l}\right)}^{1/m}$ (j=1,2,…,m) are within $\left[\frac{\mu_{c_{j}^{h}}^{\#}}{1-\mu_{c_{j}^{h}}^{\#}},\frac{1-v_{c_{j}^{h}}^{\#}}{v_{c_{j}^{h}}^{\#}}\right]$ and are multiplicatively normalized.

Similar to the transformation methods [33,34], the maximization models in (12) are integrated and converted into

(13) $$\begin{array}{l} \max\;Z=\sum_{i=1}^{n}\sum_{j=1}^{m}\left((1-\varphi_{ij}-\phi_{ij})\omega_{j}\right)\\ s.t.\{\begin{array}{ll} \sum_{j=1}^{m}\omega_{j}=1, & \\ \frac{\mu_{c_{j}^{h}}^{\#}}{1-\mu_{c_{j}^{h}}^{\#}}\leq \frac{\omega_{j}}{{\left(\prod_{l=1}^{m}\omega_{l}\right)}^{1/m}}\leq \frac{1-v_{c_{j}^{h}}^{\#}}{v_{c_{j}^{h}}^{\#}}, & j=1,2,\ldots ,m. \end{array} \end{array}$$

By substituting the optimal solution $\omega_{j}^{*}$ (j=1,2,…,m) of the model (13) into (11), an optimized comprehensive score is obtained and denoted by ${\tilde{s}}_{x_{i}}^{*}=<\mu_{x_{i}}^{*},v_{x_{i}}^{*}>$ for every i=1,2,…,n.

**Theorem** **2.** *The optimized comprehensive scores*${\tilde{s}}_{x_{i}}^{*}=<\mu_{x_{i}}^{*},v_{x_{i}}^{*}>$*(*i=1,2,…,n*) are multiplicatively normalized, i.e.,*$\prod_{i=1}^{n}\frac{\mu_{x_{i}}^{*}(1-v_{x_{i}}^{*})}{v_{x_{i}}^{*}(1-\mu_{x_{i}}^{*})}=1$.

**Proof.**  Since the IFV based vector ${\left({\tilde{a}}_{1j},{\tilde{a}}_{2j},\ldots ,{\tilde{a}}_{nj}\right)}^{T}$ is multiplicatively normalized for every j=1,2,…,m, we have $\prod_{i=1}^{n}\frac{\phi_{ij}(1-\varphi_{ij})}{\varphi_{ij}(1-\phi_{ij})}=1,\forall j=1,2,\ldots ,n$. Thus, one can get

$$\prod_{i=1}^{n}\frac{\mu_{x_{i}}^{*}(1-v_{x_{i}}^{*})}{v_{x_{i}}^{*}(1-\mu_{x_{i}}^{*})}=\prod_{i=1}^{n}{\prod_{j=1}^{m}\left(\frac{\phi_{ij}(1-\varphi_{ij})}{\varphi_{ij}(1-\phi_{ij})}\right)}^{\omega_{j}^{*}}=\prod_{j=1}^{m}{\left(\prod_{i=1}^{n}\frac{\phi_{ij}(1-\varphi_{ij})}{\varphi_{ij}(1-\phi_{ij})}\right)}^{\omega_{j}^{*}}=1.$$

This completes the proof of Theorem 2. □

Based on ${\tilde{s}}_{x_{i}}^{*}=<\mu_{x_{i}}^{*},v_{x_{i}}^{*}>$ (i=1,2,…,n), a possibility degree matrix is established as $P={\left(p_{ij}\right)}_{n\times n}={\left(p({\tilde{s}}_{x_{i}}^{*}\geq {\tilde{s}}_{x_{j}}^{*})\right)}_{n\times n}$, where $p({\tilde{s}}_{x_{i}}^{*}\geq {\tilde{s}}_{x_{j}}^{*})$ is the possibility degree formula [26] defined by

(14) $$\begin{array}{l} p({\tilde{s}}_{x_{i}}^{*}\geq {\tilde{s}}_{x_{j}}^{*})=\\ \frac{\max\left\{\begin{array}{l} 0,\ln(1-v_{x_{i}}^{*})-\ln v_{x_{i}}^{*}\\ -\ln\mu_{x_{j}}^{*}+\ln(1-\mu_{x_{j}}^{*}) \end{array}\right\}-\max\left\{\begin{array}{l} 0,\ln\mu_{x_{i}}^{*}-\ln(1-\mu_{x_{i}}^{*})\\ -\ln(1-v_{x_{j}}^{*})+\ln v_{x_{j}}^{*} \end{array}\right\}}{\ln(1-v_{x_{i}}^{*})-\ln(v_{x_{i}}^{*})-\ln\mu_{x_{i}}^{*}+\ln(1-\mu_{x_{i}}^{*})+\ln(1-v_{x_{j}}^{*})-\ln v_{x_{j}}^{*}-\ln\mu_{x_{j}}^{*}+\ln(1-\mu_{x_{j}}^{*})} \end{array}$$

Based on the possibility degree matrix P, for every evaluated alternative $x_{i}$, its priority index is determined as

(15) $$s_{x_{i}}=\frac{1}{n}\sum_{j=1}^{n}p_{ij},\;i=1,2,\ldots ,n.$$

According to the decreasing order of $s_{x_{i}}$ (i=1,2,…,n), a ranking of the evaluated alternatives is obtained, and the notation $x_{i}{\underline{≻}}_{p_{ij}}x_{j}$ is utilized to characterize the expression of the evaluated alternative $x_{i}$ being superior to $x_{j}$ with a possibility degree $p_{ij}$.

## 4. A Case Study of Evaluating Low-Carbon Tourism Scenic Spots

This section applies the proposed intuitionistic fuzzy hierarchical MCDM method to evaluate the performance of low-carbon tourism scenic spots located at Zhejiang Province of China.

In order to cope with global warming mainly caused by greenhouse gas emissions and with the environmental pollution, low-carbon economy has been recognized as a new and sustainable development pattern [3,8]. As a part of low-carbon economy, low-carbon tourism needs to reduce energy consumptions and carbon emissions as well as preserve the ecological environment [6,11]. Tourism scenic spots are important carriers of tourism activities and their low-carbon performances play crucial roles in the sustainable and green tourism development. Hence, for formulating the low-carbon tourism development strategy of Zhejiang Province of China, it is necessary to establish a system to evaluate the low-carbon development levels of tourism scenic spots.

Diverse factors affect the low-carbon performance of a tourism scenic spot. By referring to the relevant literature and consulting government officials and experts in the area of the low-carbon tourism research, this case study identifies four main criteria: Low-carbon economy ($c_{1}$), low-carbon facility and environment ($c_{2}$), low-carbon transportation, diet, and accommodation ($c_{3}$), and low-carbon management ($c_{4}$).

Tourism scenic spots desire to attain economical returns by providing various services to tourists. The representation of economical returns is the tourists’ consumption. On the other hand, the undesired output of tourism scenic spots is the carbon emission caused by tourists’ consumption, which results in the pressure on the eco-environment and is against the low-carbon economy. Thus, the criterion $c_{1}$ is divided into two sub-criteria: Per capita green consumption ($c_{11}$) and tourism scenic spot carbon intensity ($c_{12}$).

Tourism scenic spots should provide green public facilities and systems to reduce the energy consumption and save water resource, and develop plenty items including tourism lines, brand, and shopping to improve the satisfaction of tourists’ consumption under the condition of no increasing the expected total amount of carbon emissions. Meanwhile, tourism scenic spots should devote to the improvement of environmental quality including the air quality and the surface water quality and the reduction of the noise pollution. Thus, the criterion $c_{2}$ is measured by three sub-criteria: Low-carbon tourism items and shopping ($c_{21}$), low-carbon public facilities and systems ($c_{22}$), and environmental quality ($c_{23}$).

Transportation connects the different destinations at a tourism scenic spot. Some scenic spots have to provide the transportation service for tourists reaching various destinations that are far apart. Thus, tourism scenic spots should make the low-carbon property more prominent by carefully considering the eco-touring trail construction, greening of scenic spot roads, low-carbon vehicles, and ecological parking areas. On the other hand, tourism scenic spots should furnish the low-carbon food service and accommodation including the use of local food materials, the disposable tableware usage and the green hotel construction. Therefore, the criterion $c_{3}$ is divided into two sub-criteria: Low-carbon transportation ($c_{31}$) and low-carbon diet and accommodation ($c_{32}$).

The low-carbon development level of a tourism scenic spot is affected by the low-carbon management and education, including dissemination of low-carbon information, low-carbon knowledge training and scenic spot staffs and local residents’ low-carbon awareness. Taking into account the details of data acquisition, this case study only considers low-carbon management and dissemination ($c_{41}$) for the management criterion $c_{4}$.

The four criteria and eight sub-criteria are characterized as a hierarchical structure shown in Table 2. In the eight sub-criteria, $c_{12}$ is a cost criterion and the others are benefit criteria.

Based on the linguistic scales shown in Table 1, importance preferences between any two of the four criteria $c_{1},c_{2},c_{3}$, and $c_{4}$ are elicited by the paired comparison method and are shown in Table 3.

According to the scale conversion shown in Table 1, an intuitionistic fuzzy preference relation (IFPR) is obtained as

$${\tilde{R}}^{\left(C\right)}={\left(<\xi_{ij}^{\left(C\right)},\eta_{ij}^{\left(C\right)}>\right)}_{4\times 4}=\left(\begin{array}{cccc} <0.5,0.5> & <2/3,\;1/4> & <4/5,\;1/6> & <6/7,\;1/8>\\ <1/4,2/3> & <0.5,0.5> & <2/3,\;1/4> & <4/5,\;1/6>\\ <1/6,4/5> & <1/4,2/3> & <0.5,0.5> & <2/3,\;1/4>\\ <1/8,6/7> & <1/6,4/5> & <1/4,2/3> & <0.5,0.5> \end{array}\right)$$

As per (A2) and (A3), we have $CI\left({\tilde{R}}^{\left(C\right)}\right)=0.0136$, $\theta_{1}=1.093,\theta_{2}=1.239,\theta_{3}=1.239$ and $\theta_{4}=1.093$. In this case study, thresholds of acceptable consistency and acceptable hesitancy are set to be 0.1 and 2, respectively, i.e., $t_{c}=0.1$ and $t_{h}=2$. Hence, the IFPR ${\tilde{R}}^{\left(C\right)}$ is acceptable. According to (A4) and (A5), importance weights of the four criteria $c_{k}$ (k=1,2,3,4) with respect to decision goal are determined and they are shown in the second column of Table 4.

By (14) and (15), the four criteria $c_{k}$ (k=1,2,3,4) are ranked as $c_{1}\overset{100\%}{\underline{≻}}c_{1}\overset{100\%}{\underline{≻}}c_{3}\overset{100\%}{\underline{≻}}c_{4}$.

Similarly, an IFPR on the corresponding sub-criteria of each of the three criteria $c_{k}$ (k=1,2,3) is determined as ${\tilde{R}}^{\left(c_{k}\right)}$, where

$${\tilde{R}}^{\left(c_{1}\right)}={\left(<\xi_{ij}^{\left(c_{1}\right)},\eta_{ij}^{\left(c_{1}\right)}>\right)}_{2\times 2}=\left(\begin{array}{cc} <0.5,0.5> & <1/4,2/3>\\ <2/3,\;1/4> & <0.5,0.5> \end{array}\right),$$

$${\tilde{R}}^{\left(c_{2}\right)}={\left(<\xi_{ij}^{\left(c_{2}\right)},\eta_{ij}^{\left(c_{2}\right)}>\right)}_{3\times 3}=\left(\begin{array}{ccc} <0.5,0.5> & <1/4,\;2/3> & <2/5,\;2/5>\\ <2/3,\;1/4> & <0.5,0.5> & <2/3,\;1/4>\\ <2/5,\;2/5> & <1/4,\;2/3> & <0.5,0.5> \end{array}\right),$$

$${\tilde{R}}^{\left(c_{3}\right)}={\left(<\xi_{ij}^{\left(c_{3}\right)},\eta_{ij}^{\left(c_{3}\right)}>\right)}_{2\times 2}=\left(\begin{array}{cc} <0.5,0.5> & <2/3,\;1/4>\\ <1/4,2/3> & <0.5,0.5> \end{array}\right).$$

Because any 2×2 IFPR is consistent, the two IFPRs ${\tilde{R}}^{\left(c_{k}\right)}$ (k=1,3) are both consistent. As per (A3), we have $\theta_{1}=\theta_{2}=1.5$ for each of the two IFPRs ${\tilde{R}}^{\left(c_{k}\right)}$ (k=1,3). Thus, ${\tilde{R}}^{\left(c_{k}\right)}$ (k=1,3) are acceptable IFPRs. By (A4) and (A5), local weights of the sub-criteria $c_{k1}$ and $c_{k2}$ are obtained from ${\tilde{R}}^{\left(c_{k}\right)}$ for each k=1,3. According to (A2) and (A3), one gets $CI\left({\tilde{R}}^{\left(c_{2}\right)}\right)=0.000005$ and $\theta_{1}=1.5,\theta_{2}=1.0002,\theta_{3}=1.5$. Therefore, ${\tilde{R}}^{\left(c_{2}\right)}$ is an acceptable IFPR. As per (A4) and (A5), local weights of the three sub-criteria $c_{21},c_{22}$ and $c_{23}$ are obtained from ${\tilde{R}}^{\left(c_{2}\right)}$. These obtained local weights are listed in the corresponding row and the fourth column of Table 4. In addition, since $c_{41}$ is a single sub-criterion with respect to the upper-level criterion $c_{4}$, its local weight is set to be <0.5, 0.5>.

According to (6) and (7), the global importance weights of the eight sub-criteria are determined and they are shown in the last column of Table 4. By (14) and (15), the sub-criteria are ranked as $c_{12}\overset{100\%}{\underline{≻}}c_{22}\overset{100\%}{\underline{≻}}c_{31}\overset{81.98\%}{\underline{≻}}c_{11}\overset{100\%}{\underline{≻}}c_{23}≅c_{21}\overset{100\%}{\underline{≻}}c_{32}\overset{100\%}{\underline{≻}}c_{41}$.

Next, four tourism scenic spots denoted by $x_{1},x_{2},x_{3}$ and $x_{4}$ are selected to evaluate their low-carbon performances based on the criteria framework shown in Table 2. The units of the two the sub-criteria $c_{11}$ and $c_{12}$ are China Yuan (CNY) and kgCO_2_/ten thousands CNY, respectively. The assessments of the four tourism scenic spots with respect to $c_{11}$ and $c_{12}$ are determined according to statistical results for 2019 and shown in the second and third columns of Table 5, respectively. Assessment information of the four tourism scenic spots with respect to other sub-criteria at the lowest level is obtained from questionnaires and is given in the last six columns of Table 5.

For the sub-criteria at the lowest-level, based on Table 5, using (9) and (10) generates eight IFPRs as

$${\tilde{R}}_{c_{11}}=\left(\begin{array}{cccc} <0.5,0.5> & <0.466,\;0.515> & <0.495,\;0.484> & <0.505,\;0.474>\\ <0.515,\;0.466> & <0.5,0.5> & <0.520,\;0.461> & <0.530,\;0.450>\\ <0.484,\;0.495> & <0.461,\;0.520> & <0.5,0.5> & <0.500,\;0.479>\\ <0.474,\;0.505> & <0.450,\;0.530> & <0.479,\;0.500> & <0.5,0.5> \end{array}\right)$$

$${\tilde{R}}_{c_{12}}=\left(\begin{array}{cccc} <0.5,0.5> & <0.460,\;0.492> & <0.517,\;0.457> & <0.528,\;0.444>\\ <0.492,\;0.460> & <0.5,0.5> & <0.524,\;0.433> & <0.535,\;0.420>\\ <0.457,\;0.517> & <0.433,\;0.524> & <0.5,0.5> & <0.500,\;0.476>\\ <0.444,\;0.528> & <0.420,\;0.535> & <0.476,\;0.500> & <0.5,0.5> \end{array}\right)$$

$${\tilde{R}}_{c_{21}}=\left(\begin{array}{cccc} <0.5,0.5> & <0.458,\;0.515> & <0.559,\;0.416> & <0.521,\;0.452>\\ <0.515,\;0.458> & <0.5,0.5> & <0.586,\;0.388> & <0.548,\;0.423>\\ <0.416,\;0.559> & <0.388,\;0.586> & <0.5,0.5> & <0.449,\;0.524>\\ <0.452,\;0.521> & <0.423,\;0.548> & <0.524,\;0.449> & <0.5,0.5> \end{array}\right)$$

$${\tilde{R}}_{c_{22}}=\left(\begin{array}{cccc} <0.5,0.5> & <0.518,\;0.461> & <0.509,\;0.464> & <0.551,\;0.419>\\ <0.461,\;0.518> & <0.5,0.5> & <0.481,\;0.494> & <0.524,\;0.448>\\ <0.464,\;0.509> & <0.494,\;0.481> & <0.5,0.5> & <0.527,\;0.439>\\ <0.419,\;0.551> & <0.448,\;0.524> & <0.439,\;0.527> & <0.5,0.5> \end{array}\right)$$

$${\tilde{R}}_{c_{23}}=\left(\begin{array}{cccc} <0.5,0.5> & <0.450,\;0.510> & <0.427,\;0.541> & <0.450,\;0.510>\\ <0.510,\;0.450> & <0.5,0.5> & <0.458,\;0.512> & <0.481,\;0.481>\\ <0.541,\;0.427> & <0.512,\;0.458> & <0.5,0.5> & <0.512,\;0.458>\\ <0.510,\;0.450> & <0.481,\;0.481> & <0.458,\;0.512> & <0.5,0.5> \end{array}\right)$$

$${\tilde{R}}_{c_{31}}=\left(\begin{array}{cccc} <0.5,0.5> & <0.500,\;0.486> & <0.540,\;0.444> & <0.560,\;0.423>\\ <0.486,\;0.500> & <0.5,0.5> & <0.531,\;0.450> & <0.552,\;0.429>\\ <0.444,\;0.540> & <0.450,\;0.531> & <0.5,0.5> & <0.511,\;0.468>\\ <0.423,\;0.560> & <0.429,\;0.552> & <0.468,\;0.511> & <0.5,0.5> \end{array}\right)$$

$${\tilde{R}}_{c_{32}}=\left(\begin{array}{cccc} <0.5,0.5> & <0.486,\;0.500> & <0.515,\;0.469> & <0.531,\;0.456>\\ <0.500,\;0.486> & <0.5,0.5> & <0.521,\;0.461> & <0.537,\;0.448>\\ <0.469,\;0.515> & <0.461,\;0.521> & <0.5,0.5> & <0.507,\;0.477>\\ <0.456,\;0.531> & <0.448,\;0.537> & <0.477,\;0.507> & <0.5,0.5> \end{array}\right)$$

$${\tilde{R}}_{c_{41}}=\left(\begin{array}{cccc} <0.5,0.5> & <0.500,\;0.486> & <0.492,\;0.492> & <0.506,\;0.480>\\ <0.486,\;0.500> & <0.5,0.5> & <0.486,\;0.500> & <0.500,\;0.488>\\ <0.492,\;0.492> & <0.500,\;0.486> & <0.5,0.5> & <0.506,\;0.480>\\ <0.480,\;0.506> & <0.488,\;0.500> & <0.480,\;0.506> & <0.5,0.5> \end{array}\right)$$

For each of the generated IFPRs, using (A2) and (A3) obtains results listed in Table 6.

All consistency indices given in Table 6 are equal to 0, implying the eight generated IFPRs are all consistent. It is clear that the values of $\theta_{i}$ (i=1,2,3,4) shown in Table 6 are all smaller than the acceptable hesitancy threshold $t_{h}=2$. Therefore, the generated IFPRs are all acceptable.

According to (A4) and (A5), a multiplicatively normalized intuitionistic fuzzy decision matrix is obtained from the eight generated IFPRs. This matrix is shown in Table 7, where the eight sub-criteria and their intuitionistic fuzzy weights are displayed in the first row.

Based on (13) and Table 7, a maximization optimization model is constructed as

$$\begin{array}{l} \max\;Z=0.04\omega_{1}+0.071\omega_{2}+0.054\omega_{3}+0.054\omega_{4}+0.07\omega_{5}+0.034\omega_{6}+0.049\omega_{7}+0.028\omega_{8}\\ s.t.\{\begin{array}{l} \sum_{j=1}^{8}\omega_{j}=1,\\ 0.961\leq \omega_{1}/{\left(\prod_{j=1}^{8}\omega_{j}\right)}^{1/8}\leq 1.232,\;2.356\leq \omega_{2}/{\left(\prod_{j=1}^{8}\omega_{j}\right)}^{1/8}\leq 3.016,\\ 0.661\leq \omega_{3}/{\left(\prod_{j=1}^{8}\omega_{j}\right)}^{1/8}\leq 1.066,\;1.985\leq \omega_{4}/{\left(\prod_{j=1}^{8}\omega_{j}\right)}^{1/8}\leq 2.135,\\ 0.661\leq \omega_{5}/{\left(\prod_{j=1}^{8}\omega_{j}\right)}^{1/8}\leq 1.066,\;1.114\leq \omega_{6}/{\left(\prod_{j=1}^{8}\omega_{j}\right)}^{1/8}\leq 1.519,\\ 0.453\leq \omega_{7}/{\left(\prod_{j=1}^{8}\omega_{j}\right)}^{1/8}\leq 0.618,\;0.330\leq \omega_{8}/{\left(\prod_{j=1}^{8}\omega_{j}\right)}^{1/8}\leq 0.361. \end{array} \end{array}$$

Solving the above optimization model gets an optimal solution as

$$\begin{array}{l} \omega_{1}^{*}=0.097,\;\omega_{2}^{*}=0.305,\;\omega_{3}^{*}=0.099,\;\omega_{4}^{*}=0.200,\\ \omega_{5}^{*}=0.108,\;\omega_{6}^{*}=0.112,\;\omega_{7}^{*}=0.046,\;\omega_{8}^{*}=0.033. \end{array}$$

The above result demonstrates that under the general weighting scheme shown in the last column of Table 4, the optimal real weights of the lowest level sub-criteria are determined for this evaluation problem. This reveals that the important weight determination method for the evaluated criteria in this paper differs from any of the existing methods in [17,18,19,20,21,22,23,24].

Substituting this optimal solution into (11) obtains IFV-based comprehensive scores of the four evaluated tourism scenic spots as

$${\tilde{s}}_{x_{1}}^{*}=<0.507,0.481>,{\tilde{s}}_{x_{2}}^{*}=<0.511,0.471>,{\tilde{s}}_{x_{3}}^{*}=<0.486,0.503>,{\tilde{s}}_{x_{4}}^{*}=<0.469,0.518>.$$

According to (14) and (15), the ranking of the performances of the four evaluated low-carbon tourism scenic spots are ranked as $x_{2}\overset{73.3\%}{\underline{≻}}x_{1}\overset{100\%}{\underline{≻}}x_{3}\overset{100\%}{\underline{≻}}x_{4}$.

## 5. Conclusions

In this research, we proposed a method to tackle hierarchical MCDM problems, where the importance weights of criteria and sub-criteria are without past data and the assessments of alternatives with respect to the lowest level sub-criteria are characterized by different dimension interval values derived from statistical tables and questionnaires. A new intuitionistic fuzzy based model was developed to obtain the importance weights of the lowest level sub-criteria. We presented an approach to generate consistent IFPRs from interval-value-based assessments of alternatives with respect to each of the lowest level sub-criteria. A novel method was devised to construct a multiplicatively normalized intuitionistic fuzzy decision matrix from the generated consistent IFPRs, and an optimization model was devised for obtaining comprehensive scores of alternatives.

The case study in this paper built an evaluation framework of the performance and efficiency of low-carbon tourism scenic spots and obtained important weights of evaluation criteria. Among the first level criteria, low-carbon economy ($c_{1}$) is identified to be the most important criterion, and low-carbon facility and environment ($c_{2}$), low-carbon transportation, diet, and accommodation ($c_{3}$), and low-carbon management ($c_{4}$) are identified to be the second, third and fourth, respectively. Among the eight lowest level sub-criteria, carbon intensity ($c_{12}$), low-carbon public facilities and systems ($c_{22}$), and low-carbon transportation ($c_{31}$) are the first three pivotal sub-criteria. Furthermore, for the specific performance evaluation problem given in the case study, carbon intensity ($c_{12}$) had a real-valued importance weight of 0.305 (the largest weight). Low-carbon public facilities and systems ($c_{22}$) had a real-valued importance weight of 0.2. Low-carbon transportation ($c_{31}$) had a real-valued importance weight of 0.112. Environmental quality ($c_{23}$) had a real-valued importance weight of 0.108. Low-carbon tourism items and shopping ($c_{21}$) had a real-valued importance weight of 0.099. Per capita green consumption ($c_{11}$) had a real-valued importance weight of 0.097. Low-carbon diet and accommodation ($c_{32}$) had a real-valued importance weight of 0.046. Low-carbon management and dissemination ($c_{41}$) had a real-valued importance weight of 0.033 (the smallest weight). These results can be utilized as a set of references and standards for formulating low-carbon tourism scenic spots. The obtained best low-carbon tourism scenic spot could be used as a learning benchmark. Meanwhile, the tourism sector can use the proposed evaluation framework to detect existing problems of tourism scenic spots and seek for corresponding solutions for improving their low-carbon performances.

The paired comparisons used to obtain importance weights of criteria are assumed to be all complete. Sometimes, however, some paired comparisons of an IFPR may be absent. In the future, we will focus on seeking a way to obtain priority weights of incomplete IFPRs. In addition, it would be interesting to apply the proposed hierarchical MCDM method to other areas such as the selection of environmental governance solutions, low-carbon supplier selection and public health management.

## Figures and Tables

**Table 1:** Linguistic scales and intuitionistic fuzzy scales for comparing the importance of criteria.

Linguistic Term	Intuitionistic Fuzzy Scale
Absolutely important (AI)	<8/9, 0.1>
Very strongly important (VSI)	<6/7, 1/8>
Strongly important (SI)	<4/5, 1/6>
Moderately important (MI)	<2/3, 1/4>
Just equal important (JEI)	<0.5, 0.5>
Approximate equally important (AEI)	<0.45, 0.45>
Moderately not important (MNI)	<1/4, 2/3>
Strongly not important (SNI)	<1/6, 4/5>
Very strongly not important (VSNI)	<1/8, 6/7>
Absolutely not important (AI)	<0.1, 8/9>

**Table 2:** The hierarchical structure of evaluation criteria and sub-criteria.

Criterion	Sub-Criterion	Definition	Benefit/Cost
Low-carbon economy ($c_{1}$)	Per capita green consumption ($c_{11}$)	This sub-criterion measures the consumption per tourist in the low-carbon tourism scenic spot	Benefit
	Tourism scenic spot carbon intensity ($c_{12}$)	This sub-criterion measures the carbon emission per tourist income in the tourism scenic spot	Cost
Low-carbon facility and environment ($c_{2}$)	Low-carbon tourism items and shopping ($c_{21}$)	This sub-criterion measures the development level of low-carbon tourism lines, brand and shopping.	Benefit
	Low-carbon public facilities and systems ($c_{22}$)	This sub-criterion measures the low-carbon development level of public facilities and systems, including energy-saving lightings, sorting trash cans, low-carbon toilets, low-carbon guiding signs, water-saving technique, clean energy usage and waste disposal system	Benefit
	Environmental quality ($c_{23}$)	This sub-criterion measures the environment comprehensive quality including the air quality, the surface water quality, and the noise pollution reduction and vegetation coverage ratio.	Benefit
Low-carbon transportation, diet and accommodation ($c_{3}$)	Low-carbon transportation ($c_{31}$)	This sub-criterion measures the low-carbon development level of transportation, including eco-touring trail construction, greening of scenic spot roads, low-carbon vehicles and ecological parking areas.	Benefit
	Low-carbon diet and accommodation ($c_{32}$)	This sub-criterion measures the low-carbon development level of diet and accommodation, including the use of local food materials, the disposable tableware usage, and the proportion of green hotel.	Benefit
Low-carbon management ($c_{4}$)	Low-carbon management and dissemination ($c_{41}$)	This sub-criterion measures the development level of low-carbon management and education, including dissemination of low-carbon information, low-carbon knowledge training and scenic spot staffs and local residents’ low-carbon awareness	Benefit

**Table 3:** Linguistic term based paired comparison results on criteria $c_{1},c_{2},c_{3}$, and $c_{4}$.

	$c_{1}$	$c_{2}$	$c_{3}$	$c_{4}$
$c_{1}$	JEI	MI	SI	VSI
$c_{2}$	MNI	JEI	MI	SI
$c_{3}$	SNI	MNI	JEI	MI
$c_{4}$	VSNI	SNI	MNI	JEI

**Table 4:** Importance weights of evaluation criteria and sub-criteria.

Criterion	Weight of the Criterion	Sub-Criterion	Local Weight of the Sub-Criterion	Global Importance Weight of the Sub-Criterion
$c_{1}$	<0.735, 0.248>	$c_{11}$	<0.366, 0.586>	<0.490, 0.448>
		$c_{12}$	<0.586, 0.366>	<0.702, 0.249>
$c_{2}$	<0.566, 0.382>	$c_{21}$	<0.377, 0.524>	<0.398, 0.484>
		$c_{22}$	<0.645, 0.355>	<0.665, 0.319>
		$c_{23}$	<0.377, 0.524>	<0.398, 0.484>
$c_{3}$	<0.382, 0.566>	$c_{31}$	<0.586, 0.366>	<0.527, 0.397>
		$c_{32}$	<0.366, 0.586>	<0.312, 0.618>
$c_{4}$	<0.248, 0.735>	$c_{41}$	<0.5, 0.5>	<0.248, 0.735>

**Table 5:** Assessments of the four tourism scenic spots with respect to each of the sub-criteria at the lowest level.

	$c_{11}$	$c_{12}$	$c_{21}$	$c_{22}$	$c_{23}$	$c_{31}$	$c_{32}$	$c_{41}$
$x_{1}$	[480, 500]	[1.60, 1.70]	[7.6, 8.0]	[8.6, 9.0]	[6.7, 7.3]	[8.8, 9.0]	[8.5, 8.7]	[8.7, 9.0]
$x_{2}$	[530, 550]	[1.45, 1.65]	[8.5, 9.0]	[7.7, 8.0]	[7.6, 8.2]	[8.5, 8.8]	[8.7, 9.0]	[8.5, 8.7]
$x_{3}$	[470, 490]	[1.82, 1.90]	[5.7, 6.0]	[7.8, 8.3]	[8.6, 9.0]	[7.2, 7.5]	[7.7, 8.0]	[8.7, 9.0]
$x_{4}$	[450, 470]	[1.90, 2.00]	[6.6, 7.0]	[6.5, 7.0]	[7.6, 8.2]	[6.6, 6.9]	[7.3, 7.5]	[8.3, 8.5]

**Table 6:** Consistency indices and $\theta_{i}$ values (i=1,2,3,4 ) of the generated IFPRs.

IFPR	${\tilde{R}}_{c_{11}}$	${\tilde{R}}_{c_{12}}$	${\tilde{R}}_{c_{21}}$	${\tilde{R}}_{c_{22}}$	${\tilde{R}}_{c_{23}}$	${\tilde{R}}_{c_{31}}$	${\tilde{R}}_{c_{32}}$	${\tilde{R}}_{c_{41}}$
CI(.)	0	0	0	0	0	0	0	0
$\theta_{1}$	1.042	1.063	1.052	1.048	1.089	1.024	1.025	1.033
$\theta_{2}$	1.036	1.140	1.060	1.038	1.079	1.034	1.033	1.024
$\theta_{3}$	1.042	1.044	1.052	1.064	1.046	1.042	1.039	1.033
$\theta_{4}$	1.044	1.053	1.060	1.078	1.079	1.045	1.027	1.024

**Table 7:** Multiplicatively normalized intuitionistic fuzzy decision matrix.

	$c_{11}$	$c_{12}$	$c_{21}$	$c_{22}$	$c_{23}$	$c_{31}$	$c_{32}$	$c_{41}$
	<0.490, 0.448>	<0.702, 0.249>	<0.398, 0.484>	<0.665, 0.319>	<0.398, 0.484>	<0.527, 0.397>	<0.312, 0.618>	<0.248, 0.735>
$x_{1}$	<0.494, 0.496>	<0.506, 0.478>	<0.513, 0.474>	<0.524, 0.465>	<0.460, 0.519>	<0.528, 0.466>	<0.510, 0.484>	<0.501, 0.491>
$x_{2}$	<0.519, 0.472>	<0.514, 0.454>	<0.541, 0.445>	<0.496, 0.495>	<0.491, 0.490>	<0.520, 0.472>	<0.516, 0.457>	<0.495, 0.499>
$x_{3}$	<0.489, 0.501>	<0.479, 0.511>	<0.441, 0.546>	<0.499, 0.485>	<0.522, 0.467>	<0.478, 0.512>	<0.486, 0.505>	<0.501, 0.491>
$x_{4}$	<0.478, 0.511>	<0.466, 0.521>	<0.478, 0.508>	<0.454, 0.528>	<0.491, 0.490>	<0.457, 0.533>	<0.472, 0.521>	<0.489, 0.505>

## Appendix A

(A1) $$\delta_{k}=\frac{\max\left\{{\left(p_{k}\right)}^{1/(n-2)},{\left(P\right)}^{1/(2(n-1)(n-2))}\right\}}{\max\left\{{\left(p_{k}\right)}^{1/(n-2)},{\left(P\right)}^{1/(2(n-1)(n-2))}\right\}+\min\left\{{\left(p_{k}\right)}^{1/(n-2)},{\left(P\right)}^{1/(2(n-1)(n-2))}\right\}},k=1,2,\ldots ,n,$$

where

$$p_{k}=\prod_{j=1}^{n}\frac{\left(1-\xi_{kj})(1-\eta_{kj}\right)}{\xi_{kj}\eta_{kj}},k=1,2,\ldots ,n,P=\prod_{k=1}^{n}p_{k}$$

(A2) $$CI\left(\tilde{R}\right)=\frac{1}{n(n-1)(n-2)}\sum_{i=1}^{n-1}\sum_{j=i+1}^{n}\sum_{k\neq i,j}^{n}\left(\begin{array}{l} \left|(1-\delta_{k})\xi_{ij}(1-\xi_{ik})(1-\xi_{kj})-\delta_{k}(1-\xi_{ij})\xi_{ik}\xi_{kj}\right|\\ +\left|(1-\delta_{k})\eta_{ij}(1-\eta_{ik})(1-\eta_{kj})-\delta_{k}(1-\eta_{ij})\eta_{ik}\eta_{kj}\right| \end{array}\right)$$

(A3) $$\begin{array}{l} \theta_{i}=\{\begin{array}{cc} p_{i}, & n=2\\ \frac{\delta_{i}}{1-\delta_{i}}, & n\geq 3 \end{array}\\ =\{\begin{array}{ll} p_{i}, & n=2\\ \frac{\max\left\{{\left(p_{k}\right)}^{1/(n-2)},{\left(P\right)}^{1/(2(n-1)(n-2))}\right\}}{\min\left\{{\left(p_{k}\right)}^{1/(n-2)},{\left(P\right)}^{1/(2(n-1)(n-2))}\right\}}, & n\geq 3 \end{array} \end{array},\;i=1,2,\ldots ,n.$$

(A4) $$\mu_{i}^{*}=\{\begin{array}{ll} \frac{{\left(\prod_{j=1}^{n}\xi_{ij}\right)}^{1/n}}{{\left(\prod_{j=1}^{n}\xi_{ij}\right)}^{1/n}+{\left(\prod_{j=1}^{n}\left(1-\xi_{ij}\right)\right)}^{1/n}}, & n=2\\ \frac{{\left(p_{i}\right)}^{1/(2n)}{\left(\prod_{j=1}^{n}\xi_{ij}\right)}^{1/n}}{{\left(p_{i}\right)}^{1/(2n)}{\left(\prod_{j=1}^{n}\xi_{ij}\right)}^{1/n}+{\left(\theta_{i}\right)}^{1/2}{\left(\prod_{j=1}^{n}\left(1-\xi_{ij}\right)\right)}^{1/n}}, & n\geq 3 \end{array},$$

(A5) $$v_{i}^{*}=\{\begin{array}{ll} \frac{{\left(\prod_{j=1}^{n}\eta_{ij}\right)}^{1/n}}{{\left(\prod_{j=1}^{n}\eta_{ij}\right)}^{1/n}+{\left(\prod_{j=1}^{n}\left(1-\eta_{ij}\right)\right)}^{1/n}}, & n=2\\ \frac{{\left(p_{i}\right)}^{1/(2n)}{\left(\prod_{j=1}^{n}\eta_{ij}\right)}^{1/n}}{{\left(p_{i}\right)}^{1/(2n)}{\left(\prod_{j=1}^{n}\eta_{ij}\right)}^{1/n}+{\left(\theta_{i}\right)}^{1/2}{\left(\prod_{j=1}^{n}\left(1-\eta_{ij}\right)\right)}^{1/n}}, & n\geq 3 \end{array}$$
